# Adenovirus-Mediated Gene Transfer in Mesenchymal Stem Cells Can Be Significantly Enhanced by the Cationic Polymer Polybrene

**DOI:** 10.1371/journal.pone.0092908

**Published:** 2014-03-21

**Authors:** Chen Zhao, Ningning Wu, Fang Deng, Hongmei Zhang, Ning Wang, Wenwen Zhang, Xian Chen, Sheng Wen, Junhui Zhang, Liangjun Yin, Zhan Liao, Zhonglin Zhang, Qian Zhang, Zhengjian Yan, Wei Liu, Di Wu, Jixing Ye, Youlin Deng, Guolin Zhou, Hue H. Luu, Rex C. Haydon, Weike Si, Tong-Chuan He

**Affiliations:** 1 Departments of Clinical Hematology, Cell Biology and Oncology, the Affiliated Southwest Hospital of the Third Military Medical University, Chongqing, China; 2 Molecular Oncology Laboratory, Department of Orthopaedic Surgery, The University of Chicago Medical Center, Chicago, Illinois, United States of America; 3 Stem Cell Biology and Therapy Laboratory of the Ministry of Education Key Laboratory for Pediatrics, The Children's Hospital of Chongqing Medical University, Chongqing, China; 4 Ministry of Education Key Laboratory of Diagnostic Medicine and the Affiliated Hospitals of Chongqing Medical University, Chongqing, China; 5 Department of Laboratory Medicine, the Affiliated Hospital of Bingzhou Medical University, Yantai, China; 6 Department of Orthopaedic Surgery, the Affiliated Xiang-Ya Hospital of Central South University, Changsha, China; 7 Department of Surgery, the Affiliated Zhongnan Hospital of Wuhan University, Wuhan, China; 8 School of Bioengineering, Chongqing University, Chongqing, China; Rush University Medical Center, United States of America

## Abstract

Mesenchymal stem cells (MSCs) are multipotent progenitors, which can undergo self-renewal and give rise to multi-lineages. A great deal of attentions have been paid to their potential use in regenerative medicine as potential therapeutic genes can be introduced into MSCs. Genetic manipulations in MSCs requires effective gene deliveries. Recombinant adenoviruses are widely used gene transfer vectors. We have found that although MSCs can be infected *in vitro* by adenoviruses, high virus titers are needed to achieve high efficiency. Here, we investigate if the commonly-used cationic polymer Polybrene can potentiate adenovirus-mediated transgene delivery into MSCs, such as C2C12 cells and iMEFs. Using the AdRFP adenovirus, we find that AdRFP transduction efficiency is significantly increased by Polybrene in a dose-dependent fashion peaking at 8 μg/ml in C2C12 and iMEFs cells. Quantitative luciferase assay reveals that Polybrene significantly enhances AdFLuc-mediated luciferase activity in C2C12 and iMEFs at as low as 4 μg/ml and 2 μg/ml, respectively. FACS analysis indicates that Polybrene (at 4 μg/ml) increases the percentage of RFP-positive cells by approximately 430 folds in AdRFP-transduced iMEFs, suggesting Polybrene may increase adenovirus infection efficiency. Furthermore, Polybrene can enhance AdBMP9-induced osteogenic differentiation of MSCs as early osteogenic marker alkaline phosphatase activity can be increased more than 73 folds by Polybrene (4 μg/ml) in AdBMP9-transduced iMEFs. No cytotoxicity was observed in C2C12 and iMEFs at Polybrene up to 40 μg/ml, which is about 10-fold higher than the effective concentration required to enhance adenovirus transduction in MSCs. Taken together, our results demonstrate that Polybrene should be routinely used as a safe, effective and inexpensive augmenting agent for adenovirus-mediated gene transfer in MSCs, as well as other types of mammalian cells.

## Introduction

Mesenchymal stem cells (MSCs) are multipotent progenitors which are able to undergo self-renewal and give rise to multi-lineages, including osteogenic, chondrogenic, and adipogenic lineages [1]–[5]. While MSCs have been isolated from numerous tissues, one of the major sources in adults is the bone marrow stromal cells [4]. Several major signaling pathways, including BMPs and Wnts, play an important role in regulating MSC proliferation and lineage-specific commitments [3], [6]–[10]. Nonetheless, molecular mechanisms governing MSC proliferation and differentiation remain to be thoroughly elucidated.

As for any sources of progenitor cells, genetic manipulations (such as transgene overexpression and/or RNAi-mediated gene expression silencing) in MSCs would require effective gene deliveries. In the case of MSCs, a great deal of attentions have been paid to their potential use in regenerative medicine, where potential therapeutic genes can be introduced into MSCs for biomaterial/tissue engineering. For example, we have found that BMP9 is one of the most potent BMPs among the 14 types of BMPs in inducing osteogenic differentiation of MSCs by regulating several important downstream targets [6], [11]–[18]. It is conceivable that BMP9 can be introduced into mesenchymal progenitor cells *in vivo* or *ex vivo* for bone regeneration to treat fracture non-union and/or to facilitate spine fusion [6], [19], [20].

Recombinant adenoviruses are one of the most commonly-used gene transfer vehicles because they can transduce a wide variety of cells and/or tissues with relatively high efficiency [21]–[24]. Adenovirus infection is mediated by the coxsackievirus-adenovirus receptor (CAR) via the knob domain of the fiber protein and the major histocompatibility complex (MHC) class Ia-2 domain at the host cell surface [21]–[23]. After the initial attachment of the virus, the penton base interacts with αvβ3 and αvβ5 integrins, leading to internalization of the virus via receptor-mediated endocytosis [21]–[23]. One of the major limitations of adenoviruses is that the sensitivity of target cells to adenoviral infection correlates with cellular CAR expression. Depending on the CAR receptor levels and other cofactors, adenovirus infection efficiency varies drastically among cell lines [22], [25], [26]. We have found that although MSCs can be infected *in vitro* by adenoviruses, high virus titers have to be used to achieve high efficiency in these progenitor cells [11], [12].

In this study, we investigate if the cationic polymer Polybrene can be used to enhance or potentiate adenovirus-mediated transgene delivery into MSCs, such as C2C12 cells and iMEFs. Polybrene is widely used to promote the efficiency of recombinant retrovirus or lentivirus infection [27]–[30]. Using the AdRFP adenovirus, we find that the AdRFP transduction efficiency is significantly increased by Polybrene in a dose-dependent fashion with a peak at 8 μg/ml in both C2C12 and iMEFs cells. Quantitative luciferase assay reveals that Polybrene significantly enhances AdFLuc-mediated luciferase activity in C2C12 and iMEFs cells at as low as 4 μg/ml and 2 μg/ml, respectively. FACS analysis indicates that Polybrene (4 μg/ml) increases the percentage of RFP-positive cells by approximately 430 folds in AdRFP-transduced iMEFs. Furthermore, we demonstrate that Polybrene can enhance AdBMP9-induced osteogenic differentiation of mesenchymal stem cells as the early osteogenic marker alkaline phosphatase (ALP) activity can be significantly increased more than 73 folds by Polybrene (4 μg/ml) in AdBMP9-transduced iMEFs cells. Cytotoxicity analysis indicates that most C2C12 and iMEFs cells are viable at the Polybrene concentrations up to 40 μg/ml, which is about 10-fold higher than the effective concentration (i.e., 4 μg/ml) required to enhance the recombinant adenovirus' transduction efficiency in mesenchymal progenitor cells. Therefore, our results strongly suggest that Polybrene may be used as a safe, effective and inexpensive enhancer to augment adenovirus-mediated gene transfers in MSCs.

## Materials and Methods

### Cell culture and chemicals

Mouse preosteoblastic progenitor C2C12 cells were purchased from ATCC (Manassas, VA). The iMEFs were the immortalized mouse embryonic fibroblasts as previously described [31]. The cell lines were maintained in completed DMEM as described [32]–[35]. Polybrene (hexadimethrine bromide) was obtained from Sigma-Aldrich (St. Louis, MO). Unless indicated otherwise, all chemicals were purchased from Sigma-Aldrich or Fisher Scientific (Pittsburgh, PA).

### Construction and generation of recombinant adenovirus expressing RFP, FLuc, or BMP9

Recombinant adenoviruses were generated using the AdEasy technology as described [11], [12], [36]–[38]. The coding regions of monomeric red fluorescent protein (RFP), firefly luciferase (FLuc), and human BMP9 were PCR amplified and cloned into adenoviral shuttle vectors, which were subsequently used to generate recombinant adenoviruses in HEK-293 cells as described [36], [38]. The amplified adenoviruses were titrated and stored at −80°C.

### Crystal violet cell proliferation assay

Crystal violet stain assay was carried out as previously described [34], [39]–[41]. Briefly, subconfluent C2C12 and iMEFs cells were treated with different concentrations of Polybrene. At 4 days after treatment, the cells were fixed and subjected to Crystal violet staining. For the quantitative analysis, the stained cells were dissolved in 10% acetic acid and subjected to the measurement of absorbance at 570–590 nm [40], [41]. Each assay condition was done in triplicate.

### Flow cytometry analysis

Subconfluent C2C12 and iMEFs cells were infected with AdRFP and treated with different concentrations of Polybrene. At 48 h post infection the cells were collected, washed with PBS, and subjected to FACS analysis of RFP-positive cells using the BD LSR II Flow Cytometer and the FlowJo software. Uninfected cells were used as negative controls. Each assay condition was done in triplicate.

### Luciferase reporter assay

Firefly luciferase reporter assay was carried out as previously described [17], [42]–[45]. Briefly, subconfluent C2C12 and iMEFs cells were infected with AdFLuc and treated with different concentrations of Polybrene. At 24 h and 48 h post infection cells were lysed and collected for firefly luciferase activity assay using the Luciferase Assay Kit (Promega, Madison, WI). Each treatment/assay condition was performed in triplicate.

### Alkaline phosphatase (ALP) activity assays

ALP activity assays were conducted as described [11], [12], [31]–[33], [37], [44], [46], [47]. For the quantitative analysis, the ALP activity was assessed with a modified assay using the Great Escape SEAP Chemiluminescence assay kit (BD Clontech, Mountain View, CA). For qualitative assay, the ALP activity was histochemically stained using a mixture of 0.1 mg/ml napthol AS-MX phosphate and 0.6 mg/ml Fast Blue BB salt. Each assay condition was performed in triplicate, and the results were repeated in at least three independent experiments.

### Statistical analysis

The quantitative experiments were performed in triplicate and/or repeated three times. Data were expressed as mean ±SD. Statistical significances between groups were determined by one-way analysis of variance and the Student's *t* test. A value of *p*<0.05 was considered statistically significant.

## Results and Discussion

### Adenovirus-mediated RFP expression in mesenchymal progenitor cells is enhanced by Polybrene in a dose-dependent fashion

Recombinant adenoviruses are one of the most widely used gene delivery systems for *in vitro* and *in vivo* studies. While many types of mammalian cells can be transduced effectively by adenoviral vectors, the infection efficacy varies drastically among cell types/lines, largely due to the expression levels of CAR receptors. We have found that, while adenoviruses can infect mesenchymal stem cells (MSCs), significantly higher titers (e.g., multiplicities of infection, MOI at 20–100) to achieve high infection efficiency [11], [12].

**Figure 1:**
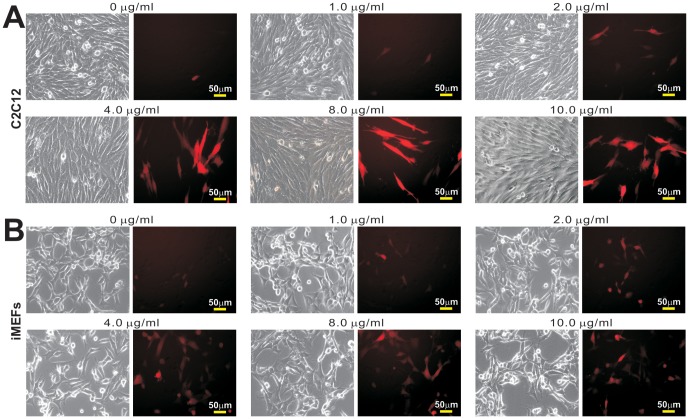
Polybrene enhances adenovirus-mediated RFP expression in mesenchymal progenitor cells in a dose-dependent fashion. Subconfluent C2C12 (**A**) and iMEFs (**B**) cells were infected with AdRFP at a multiplicity of infection (MOI) = 5. Various concentrations of Polybrene were at the time of infection. At 48 h post infection RFP expression was recorded under a fluorescence microscope with the same exposure time and sensitivity. Each infection/treatment condition was done in duplicate. Representative results are shown.

Here we analyzed if Polybrene, a commonly-used polycation for retroviral infection, would potentiate adenovirus-mediated gene transduction in MSCs, such as C2C12 and iMEFs. We found that subconfluent C2C12 cells were barely transduced by AdRFP at the MOI of 5 (**Figure 1A**). However, the AdRFP transduction efficiency was significantly increased in the presence of Polybrene and responded in a dose-dependent fashion with a peak at 8 μg/ml (**Figure 1A**). In iMEFs cells, we found that the AdRFP infectivity was extremely low at the MOI of 5, which was significantly enhanced by Polybrene (**Figure 1B**). Thus, these results strongly suggest that Polybrene may enhance adenovirus-mediated gene transduction.

### AdFLuc-mediated firefly luciferase activity in mesenchymal progenitor cells is significantly augmented by Polybrene

**Figure 2:**
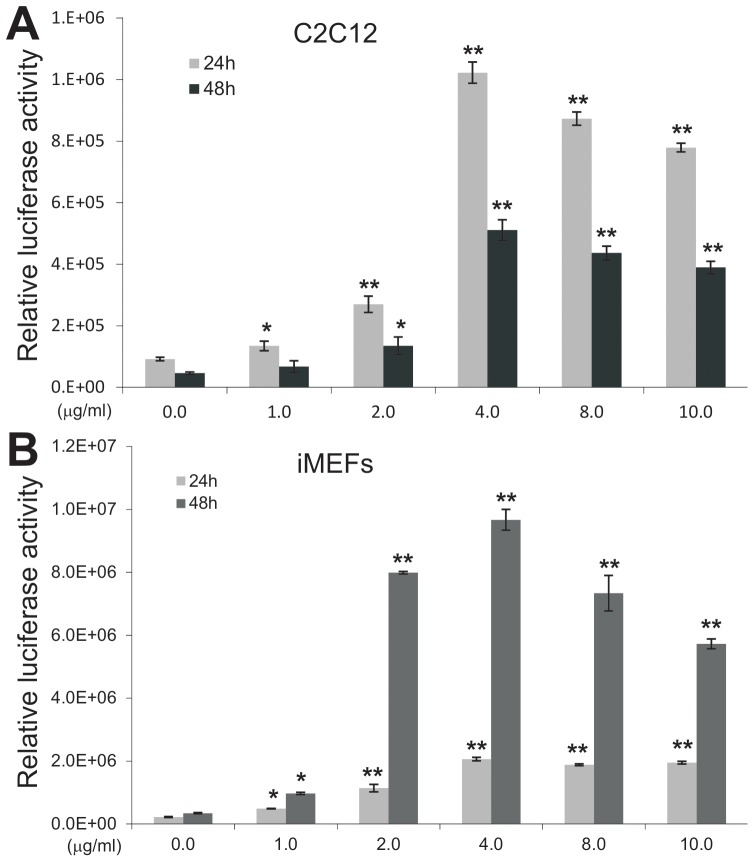
Polybrene enhances adenovirus-mediated firefly luciferase activity in mesenchymal progenitor cells. Subconfluent C2C12 (**A**) and iMEFs (**B**) cells were infected with AdFLuc at a multiplicity of infection (MOI) = 5. Various concentrations of Polybrene were added at the time of infection. At 24 h and 48 h post infection cells were lysed and collected for firefly luciferase activity assays. Each treatment condition was done in triplicate. “*”, p<0.05; “**”, p<0.001.

We next quantitatively determined the effect of Polybrene on adenovirus-mediated transgene expression using the AdFLuc vector. When C2C12 cells were seeded at the same condition and infected with the same titer of AdFLuc, the addition of Polybrene at the time of infection significantly increased the luciferase activities in a dose-dependent fashion both at 24 h and 48 h after infection, which peaked at 4 μg/ml and maintained plateaued up to 10 μg/ml (**Figure 2A**). Similar results were obtained in iMEFs except that the Polybrene-enhanced luciferase activities were more pronounced at 48 h after infection (**Figure 2B**). In addition, it seems that Polybrene enhanced luciferase activities in iMEFs significantly at a lower concentration (2 μg/ml) than that in C2C12 cells, although peaked at a similar concentration (4 μg/ml). Taken together, these quantitative results further demonstrate that adenovirus-mediated transgene transduction can be markedly enhanced by inclusion of Polybrene at the time of infection.

### FACS analysis reveals that Polybrene increases adenovirus infection efficiency in AdRFP transduced mesenchymal stem cells

**Figure 3:**
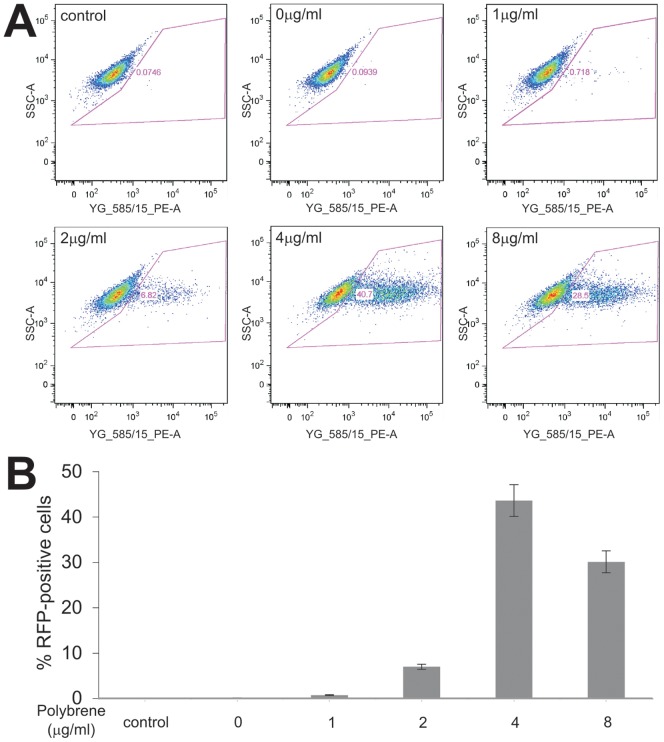
FACS analysis of Polybrene enhanced adenovirus-mediated RFP expression in mesenchymal stem cells. Subconfluent iMEFs cells were infected with AdRFP at a multiplicity of infection (MOI) = 5. Various concentrations of Polybrene were added at the time of infection. At 48 h post infection the cells were collected and subjected to FACS analysis of RFP-positive cells. Representative results are shown (**A**). Each treatment condition was done in triplicate and graphed (**B**).

In order to determine if Polybrene-enhanced luciferase activity is caused by increasing the transgene expression and/or adenovirus infection efficiency, we used FACS analysis to evaluate if Polybrene treatment increased the numbers of RFP-positive cells. The iMEFs cells were infected with the same titer of AdRFP and treated with varied concentrations of Polybrene. When the cells were collected and subjected to FACS analysis, we found that the percentages of RFP-positive cells were significantly increased by Polybrene in a dose-dependent fashion (**Figure 3A**). Quantitatively, the average % of RFP-positive cells increased from 0.1% at 0 μg/ml to 43.6% at 4 μg/ml Polybrene, approximately 430-fold increase in the percentage of RFP-positive cells although no significant further increase at concentrations higher than 8 μg/ml (**Figure 3B**). These results strongly suggest that Polybrene may significantly improve adenovirus infection efficiency in addition to promoting transgene expression in mesenchymal stem cells.

### AdBMP9-induced osteogenic differentiation of mesenchymal stem cells is significantly enhanced by Polybrene

**Figure 4:**
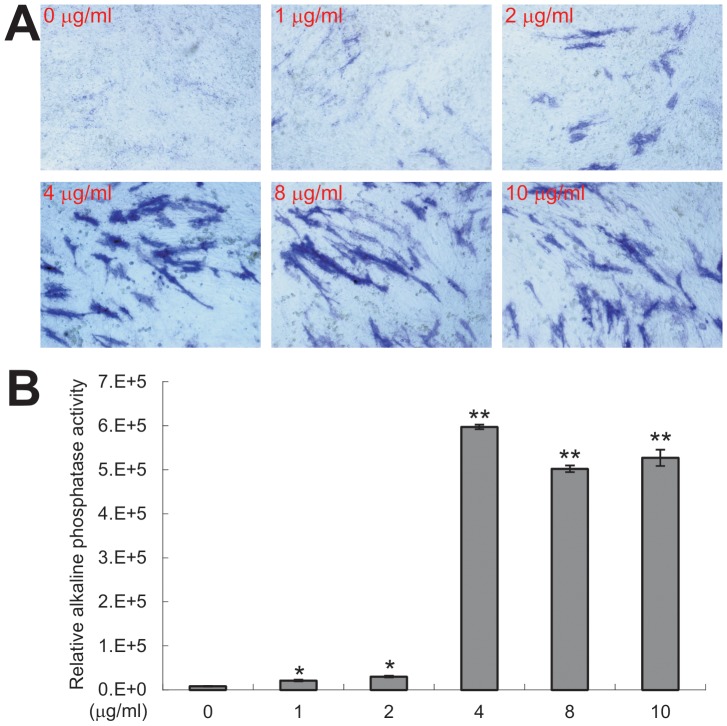
Polybrene enhances AdBMP9 transduction and osteogenic differentiation in MSCs. Subconfluent iMEFs cells were infected with AdBMP9 (or AdRFP, not shown) at a multiplicity of infection (MOI) = 5. Various concentrations of Polybrene were added at the time of infection. At 5 days post infection the cells were subjected to qualitative histochemical staining (**A**) or quantitative chemiluminescence assay (**B**). Each treatment condition was done in triplicate. Representative results are shown. “*”, p<0.05; “**”, p<0.001.

We previously demonstrated that BMP9 is one of the most potent osteogenic factors to induce osteogenic differentiation *in vitro* and *in vivo* [6], [11], [12], [18], [20], [48]. Here, we conducted further functional assays to determine if Polybrene can enhance AdBMP9-induced osteogenic differentiation of mesenchymal stem cells. When iMEFs were infected with the same titer of AdBMP9 and treated with different concentrations of Polybrene, we found that the early osteogenic marker alkaline phosphatase (ALP) activity increased in the presence of Polybrene in a dose-dependent fashion, which peaked at 4 μg/ml as shown by qualitative histochemical staining (**Figure 4A**). Quantitatively, the ALP activities were shown to increase by approximately 2.6, 3.7, 73.6, 61.9 and 65.0-fold in the presence of 1, 2, 4, 8, and 10 μg/ml of Polybrene, respectively, compared with that of the no Polybrene group (**Figure 4B**). Polybrene itself did not induce any detectable ALP activity (data not shown). These results demonstrate that Polybrene at as low as 4 μg/ml can significantly enhance the ability of AdBMP9 virus to transduce iMEFs and hence induce osteogenic differentiation of mesenchymal stem cells.

### Mesenchymal progenitor cells can tolerate a broad range of concentrations of Polybrene

**Figure 5:**
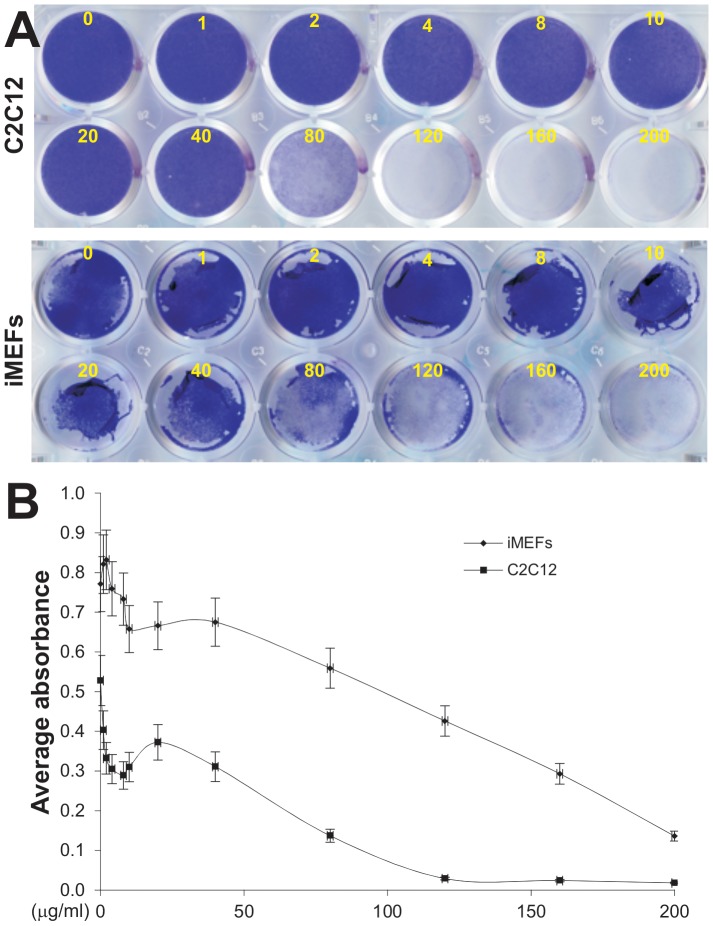
Cytoxicity of Polybrene in mesenchymal progenitor cells. (**A**) Crystal violet staining. Subconfluent C2C12 and iMEFs cells were treated with various concentrations of Polybrene. At 4 days after treatment, the cells were fixed and subjected to Crystal violet staining. Each treatment condition was done in triplicate. Representative results are shown. (**B**) Quantitative analysis of the Crystal violet-stained cells. The stained cells from (**A**) were dissolved in 10% acetic acid and subjected to the measurement of absorbance at 570–590 nm. Each assay condition was done in triplicate.

Lastly, we sought to test the cytotoxic effect of Polybrene on mesenchymal progenitor cells. When subconfluent C2C12 and iMEFs cells were treated with various concentrations of Polybrene and stained with Crystal violet, we found that most C2C12 cells and iMEFs remained viable at the Polybrene concentrations up to 40 μg/ml (**Figure 5A**), which is about 10-fold higher than the effective concentration (i.e., 4 μg/ml) required to enhance adenovirus transduction efficiency in mesenchymal progenitor cells. Quantitative analysis of the Crystal violet-stained cells revealed a similar trend although significant toxicities were apparent when Polybrene concentrations were at 80 μg/ml or higher (**Figure 5B**). These data indicate that Polybrene is well tolerated by mesenchymal progenitor cells, and most importantly, that there is a relatively large window between the effective concentration and toxicity concentration.

### Polybrene is a safe, effective and inexpensive cationic polymer that can be used to augment adenovirus-mediated gene transfer

As a cationic polymer Polybrene is widely used to increase the efficiency of retrovirus infection of certain cells *in vitro* [27]–[30]. It has been postulated that Polybrene acts by neutralizing the charge repulsion between virions and sialic acid on the cell surface [49]. Several studies showed that Polybrene, together with dimethyl sulfoxide, can be used to mediate DNA transfection in many types of cells, including CHO cells [50]–[54]. Our fluorescence microscopic results and FACS analysis strongly suggest that Polybrene may augment the adenovirus-mediated gene transfer by enhancing both infection efficiency and transgene expression. Nonetheless, the exact mechanisms through which Polybrene enhances adenovirus-mediated transgene expression remain to be fully understood.

An earlier study examined the use of polycations, such as Polybrene, protamine, DEAE-dextran, and poly-L-lysine, to enhance adenovirus type 2 vector-mediated gene delivery in primary cultures of human airway, Madin-Darby canine kidney cells, an immortalized cystic fibrosis airway epithelial cell line, and primary cultures of sheep pulmonary artery endothelium and found that these polycations increased the percentage of Ad2-transduced cells [55]. It was also shown that in human and rat epithelial cell lines and keratinocytes the addition of Polybrene during adenoviral transduction of Adβ-gal resulted in a marked increase of β-gal positive cells and β-gal protein expression in dose-dependent manner [56]. A recent study reported that Polybrene can improve transduction efficacy of recombinant adenovirus in cutaneous cells and burned skin models [57]. These findings in epithelial and endothelial cells are consistent with our results obtained from mesenchymal stem cells. Thus, these findings strongly suggest that Polybrene may be used an universal enhancer for recombinant adenovirus infection of mammalian cells. Interestingly, the polyanion heparin did not significantly affect adenovirus-mediated gene transfer efficiency, but completely abrogated the effects of polycations [55], suggesting that negatively charged moieties on the cell surface reduce the efficiency of adenovirus-mediated gene transfer, and that alteration of the charge interaction between adenoviruses and the cell surface may improve adenovirus infection efficiency.

In summary, we investigate if Polybrene can enhance adenovirus-mediated transgene delivery into MSCs lines C2C12 and iMEFs. We find that AdRFP transduction efficiency is significantly increased by Polybrene in a dose-dependent fashion with a peak at 8 μg/ml in C2C12 and iMEFs cells. AdFLuc-mediated luciferase activity is enhanced by Polybrene in C2C12 and iMEFs at as low as 4 μg/ml and 2 μg/ml, respectively. FACS analysis indicates that Polybrene (4 μg/ml) increases the percentage of RFP-positive cells by approximately 430 folds in AdRFP-transduced iMEFs. Furthermore, Polybrene can enhance AdBMP9-induced osteogenic differentiation of MSCs as the early osteogenic marker ALP activity can be significantly increased more than 73 folds by Polybrene (4 μg/ml) in AdBMP9-transduced iMEFs cells. Cytotoxicity analysis indicates that most C2C12 and iMEFs cells are viable at the Polybrene concentrations up to 40 μg/ml, which is about 10-fold higher than the effective concentration (i.e., 4 μg/ml) required to enhance adenovirus transduction efficiency in MSCs. Therefore, our results strongly suggest that Polybrene may be routinely used as an effective, safe, and cost-effective enhancer to augment adenovirus-mediated gene transfers in MSCs and other mammalian cells.
